# Case report of surgical treatment of scoliosis caused by neurofibroma located posterior mediastinum

**DOI:** 10.1016/j.ijscr.2018.10.071

**Published:** 2018-11-01

**Authors:** Nozomu Motono, Masahito Kawaguchi, Norio Kawahara, Hidetaka Uramoto

**Affiliations:** a Department of Thoracic Surgery, Kanazawa Medical University, Ishikawa, Japan; b Department of Orthopedic Surgery, Kanazawa Medical University, Ishikawa, Japan

**Keywords:** Neurofibromatosis type 1, Scoliosis, Posterior mediastinum, Spinal fusion, Case report

## Abstract

**Background:**

Neurofibromatosis type 1 (NF-1) is often associated with various orthopedic disorders, especially scoliosis. Spinal deformity in patients with NF-1 can be induced by localized neurofibromas.

**Case presentation:**

Scoliosis can be induced by a localized neurofibroma. A 12-year-old boy diagnosed with neurofibromatosis type 1 had severe scoliosis caused by a neurofibroma located in the posterior mediastinum. We performed two-stage procedure involving the extirpation of the neurofibroma in the lateral position, and posterior spinal fusion with segmental spinal instrumentation for the scoliosis in the prone position.

**Discussion:**

The optimum surgical approach for the extirpation of neurofibroma located in the posterior mediastinal remains to be established. It may be difficult to extirpate a tumor in this position via a posterior approach alone, as the tumor is surrounded by several tissue, e.g. azygos vein, trachea and esophagus. In our case, we first extirpated the tumor located in the posterior mediastinum via a posterolateral incision in the right lateral position. With this approach, it may be easy to confirm the safe relationship between the tumor and surrounding tissue.

**Conclusions:**

We safely performed a two-stage procedure involving the extirpation of a neurofibroma in the lateral position, and posterior spinal fusion with segmental spinal instrumentation for scoliosis in the prone position.

## Highlights

• Neurofibromatosis type 1 (NF-1) is often associated with various orthopedic disorders, especially scoliosis.
• Spinal deformity in patients with NF-1 can be induced by localized neurofibromas.
• The optimum surgical approach for the release of neurofibroma located in the posterior mediastinal remains to be established.
• We safety performed a two-stage procedure involving the release of a neurofibroma in the lateral position/extirpation of the neurofibroma and posterior spinal fusion with segmental spinal instrumentation for scoliosis in the prone position.

## 1 Introduction

Neurofibromatosis type 1 (NF-1) is often associated with various orthopedic disorders, especially scoliosis [1]. Spinal deformity in patients with NF-1 can be induced by localized neurofibromas [2,3]. Although the surgical management of spinal deformities in patients with NF-1 is a major challenge, several studies have shown the efficacy and safety of such surgical treatment in patients with NF-1 [[4], [5], [6], [7], [8], [9]].

We herein report the extirpation of neurofibroma and posterior spinal fusion with segmental spinal instrumentation in a case of scoliosis with NF-1.

This work has been reported in line with the SCARE criteria [10].

## 2 Report

**Fig. 1:**
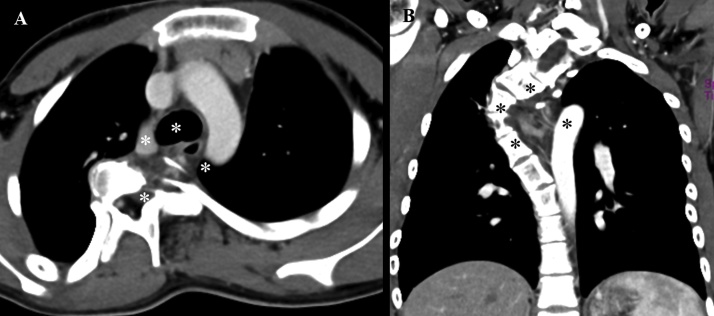
(A) Computed tomography (CT) showing the tumor located in the posterior mediastinum (white asterisks). (B) CT showing scoliosis with the tumor (black asterisks).

A 12-year-old boy was admitted to our hospital for the treatment of scoliosis. He had been diagnosed with NF-1 because of café-au-lait macules and scoliosis. The scoliotic curve measurement by Cobb’s technique was 73° on plain X-ray. Chest computed tomography revealed a neurofibroma in the posterior mediastinum (Fig. 1). We suspected that the scoliosis had been induced by the neurofibroma, so we planned a two-stage procedure involving the extirpation of the neurofibroma in the lateral position, and posterior spinal fusion with segmental spinal instrumentation for the scoliosis in the prone position.

**Fig. 2:**
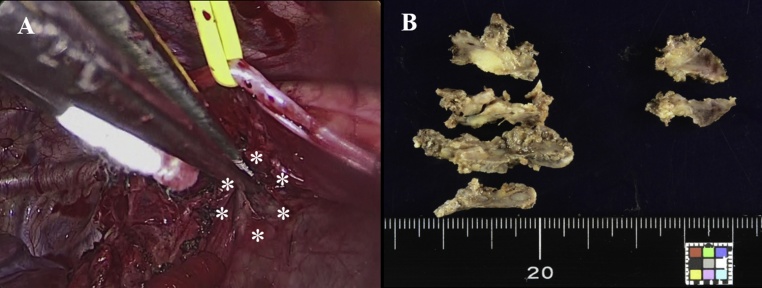
(A) Intraoperative findings showing the tumor’s dark-red appearance, being located widely adjacent to the spine and surrounding tissues (white asterisks). (B) Macroscopic findings showing that the boundary between the tumor and surrounding tissues is unclear.

**Fig. 3:**
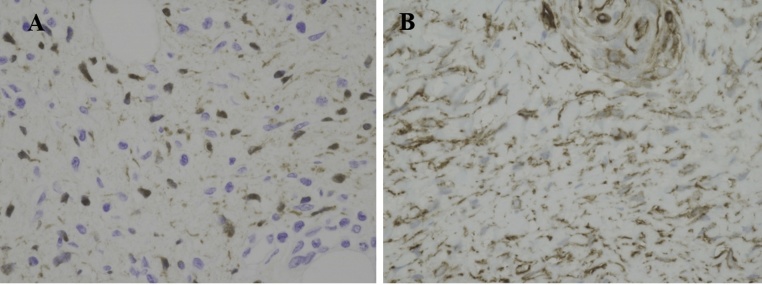
(A) Immunohistochemical staining showing the tumor stained by S-100. (B) Immunohistochemical staining showing the tumor stained by CD34.

**Fig. 4:**
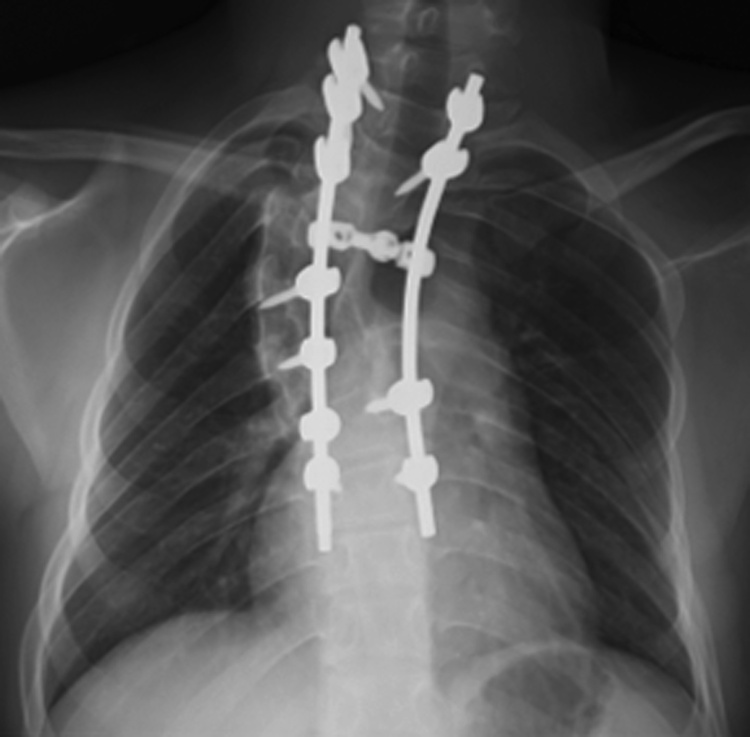
Postoperative plain X-ray showing improvement in the scoliosis.

First, extirpation of the neurofibroma was performed by a posterolateral incision in the right lateral position (Fig. 2A). The tumor appeared dark-red in color and was widely adjacent to the spine and surrounding tissues, e.g. the azygos vein and trachea. We extirpated the tumor from the surrounding tissues without involving the spine. Posterior spinal fusion with segmental spinal instrumentation for scoliosis were then performed by a middle incision in the prone position (Fig. 2B). Pathological examination revealed that the tumor was stained by S-100 and CD34, therefore the tumor was diagnosed with neurofibroma (Fig. 3A, B). The postoperative scoliotic curve measurement by Cobb’s technique was 45° (Fig. 4).

A pathological examination revealed that Schwann cells and fibroblasts had increased in numbers and infiltrated the surrounding tissues, and the tumor was diagnosed as neurofibroma.

## 3 Discussion

Scoliosis is the most common skeletal deformity of NF-1, with an occurrence of 10%–64% [2]. The optimum treatment for scoliosis in NF-1 patients remains controversial. Generally, a dystrophic scoliotic curve <20° should be closely observed for progression at 6-month intervals. For patients with scoliotic curves of ≥20°, posterior spinal fusion with segmental spinal instrumentation is indicated [[1], [2], [3], [4], [5], [6], [7], [8], [9]]. In our case, surgical intervention was deemed necessary because the frontal scoliotic curve was 73°.

Some 2%–3% of all scoliotic patients with severe curves reportedly have neurofibromatosis [1]. Aetiological theories suggest that the development of a spinal deformity in patients with neurofibromatosis is due to the erosion or infiltration of bone by localized neurofibromas, primary mesodermal dysplasia, osteomalacia and endocrine disturbances [[1], [2], [3]]. In our case, a pathological examination revealed the infiltration of tumor cells into the surrounding tissues. This suggests that neurofibroma induce spinal deformity by infiltrating the bone.

The optimum surgical approach for the extirpation of neurofibroma located in the posterior mediastinum remains to be established. It may be difficult to extirpate a tumor in this position via a posterior approach alone, as the tumor is surrounded by several tissues, e.g. azygos vein, trachea and esophagus. In our case, we first extirpated the tumor located in the posterior mediastinum via a posterolateral incision in the right lateral position. With this approach, it may be easy to confirm the safe relationship between the tumor and surrounding tissue. Extirpation of tumors located in the posterior mediastinum and posterior spinal fusion with segmental spinal instrumentation for scoliosis may need to be performed in cooperation with a thoracic surgeon and orthopedic surgeon.

## 4 Conclusion

We safely performed a two-stage procedure involving the extirpation of a neurofibroma in the lateral position, and posterior spinal fusion with segmental spinal instrumentation for scoliosis in the prone position. Surgical intervention for scoliosis caused by neurofibroma should be performed in cooperation with a thoracic surgeon and orthopedic surgeon.

## Conflicts of interest

The authors declare that they have no conflicts of interest

## Funding

This research did not receive any specific grant from funding agencies in the public, commercial, or not-for-profit sectors.

## Ethical approval

I certify that this kind of manuscript does not require ethical approval by the Ethical Committee of our institution.

## Consent

Written informed consent for publication of his clinical details and clinical images was obtained from the patient and his parents. A copy of the consent form is available for review by the Editor of this journal on request.

## Author contribution

Nozomu Motono, data collection and writing of manuscript.

Masahito Kawaguchi, data collection and writing of manuscript.

Norio Kawahara, writing and revision of manuscript.

Hidetaka Uramoto, writing and revision of manuscript.

## Registration of research studies

Surgical treatment of scoliosis caused by neurofibroma located posterior mediastinum: Case report.

UIN: researchregistry3583.

## Guarantor

Nozomu Motono.

## Provenance and peer review

Not commissioned, externally peer reviewed.
